# An Interference-Aware Traffic-Priority-Based Link Scheduling Algorithm for Interference Mitigation in Multiple Wireless Body Area Networks

**DOI:** 10.3390/s16122190

**Published:** 2016-12-20

**Authors:** Thien T. T. Le, Sangman Moh

**Affiliations:** Department of Computer Engineering, Chosun University, 309 Pilmun-daero, Dong-gu, Gwangju 61452, Korea; thanhthien92003@yahoo.com

**Keywords:** wireless body area network, link scheduling, medium access control, interference mitigation, traffic priority, optimization

## Abstract

Currently, wireless body area networks (WBANs) are effectively used for health monitoring services. However, in cases where WBANs are densely deployed, interference among WBANs can cause serious degradation of network performance and reliability. Inter-WBAN interference can be reduced by scheduling the communication links of interfering WBANs. In this paper, we propose an interference-aware traffic-priority-based link scheduling (ITLS) algorithm to overcome inter-WBAN interference in densely deployed WBANs. First, we model a network with multiple WBANs as an interference graph where node-level interference and traffic priority are taken into account. Second, we formulate link scheduling for multiple WBANs as an optimization model where the objective is to maximize the throughput of the entire network while ensuring the traffic priority of sensor nodes. Finally, we propose the ITLS algorithm for multiple WBANs on the basis of the optimization model. High spatial reuse is also achieved in the proposed ITLS algorithm. The proposed ITLS achieves high spatial reuse while considering traffic priority, packet length, and the number of interfered sensor nodes. Our simulation results show that the proposed ITLS significantly increases spatial reuse and network throughput with lower delay by mitigating inter-WBAN interference.

## 1. Introduction

With the development of recent technology in wireless networks and advanced sensors, ubiquitous health monitoring has been developed to allow wireless wearable sensors to collect the biological signals of the human body [1]. Wireless body area networks (WBANs) consist of a coordinator and multiple wireless sensors [2,3]. The coordinator collects data from the sensors and sends the data to a given healthcare monitoring system. The two standards of IEEE 802.15.4 [4] and IEEE 802.15.6 [2] can be easily used for WBANs. Originally, the IEEE 802.15.4 standard defined the physical (PHY) and medium access control (MAC) specifications for low-rate wireless personal area networks at short range (up to 100 m). On the other hand, the IEEE 802.15.6 standard defines the PHY and MAC layers for WBANs in short-range wireless communication within, on, or around the human body.

Because WBANs are relevant to medical and non-medical applications [5,6,7,8], the reliability as well as performance of WBANs is critical in public environments, such as hospitals or bus stops, where many people wear WBANs [9]. As described in reference [2], the coexistence environment of WBANs is varied from time to time by the mobility of WBANs, the number of traffic flows, and the dynamics of network topology. As a consequence, the transmission ranges can be overlapped among multiple WBANs, and the intra-WBAN communication will be interfered by the nearby WBANs, resulting in the degradation of network performance and reliability [10]. On the other hand, the quality of service (QoS) of WBAN applications is an emerging issue which can be related to different vital signals from sensor nodes. For example, various sensors of electrocardiograms (ECG), electromyography (EMG), electroencephalogram (EEG), accelerometers, heart rate, and temperature can be used, which have different characteristics of latency, packet length, and data rate [11]. The traffic generated by biomedical sensors can be categorized into on-demand, emergency, and normal traffic for medical and non-medical applications [7]. The typical values of the important parameters such as data rate and latency for various applications are summarized in Table 1. In the IEEE 802.15.6 standard, QoS is mapped into the traffic priority according to the application type of sensor nodes as in Table 2. Hence, it is necessary to ensure the quality and reliability of signals at each WBAN by mitigating inter-network interference. Furthermore, some MAC protocols schedule the packet transmission of multiple WBANs on the basis of QoS and traffic priority both for overcoming the performance degradation caused by interference and for ensuring the requirement of traffic priority [12,13].

**Figure 1:**
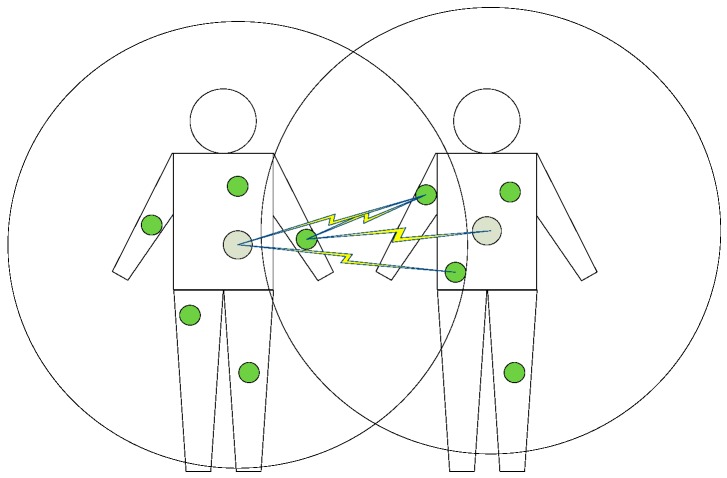
Two interfered WBANs and their interference links.

Figure 1 shows a network model of interfered WBANs, in which each person wears a WBAN. The inter-WBAN interference links are illustrated in Figure 1, and they are the links between the coordinator of a WBAN and the sensor nodes of the other WBAN. In Figure 1, the two circles represent the transmission range of the two WBANs, respectively.

Many existing interference mitigation schemes for WBANs focus on either controlling the transmission power or scheduling the working channel [14]. The transmission power of transmitting nodes is controlled to mitigate interference, in which each power controller can adapt the transmission power according to the dynamic changes of network topology [15,16,17]. The working channel of WBANs can be scheduled in time or frequency domain. For example, time division multiple access (TDMA), spatial-time division multiple access (STDMA), or frequency division multiple access (FDMA) can be used. However, it is very important to consider the traffic priority and reliability of the vital signals of the human body before scheduling the working channel in multiple WBANs.

In this paper, we focus on scheduling transmissions in multiple WBANs in the space–time domain and on maximizing network throughput with lower delay. The proposed link scheduling algorithm is run in association with the TDMA-based MAC protocol at each coordinator. We consider the number of interfered sensor nodes in each WBAN as well as the traffic priority and packet length of each type of traffic. The proposed interference-aware traffic-priority-based link scheduling (ITLS) algorithm can effectively overcome inter-WBAN interference in densely deployed WBANs, thus achieving high spatial reuse. According to the performance study, ITLS significantly increases spatial reuse and network throughput with lower delay by mitigating inter-WBAN interference.

The contributions of this work are as follows: first, we create an interference graph where the vertices represent WBANs and the edges represent the interference links. We consider the number of interfering WBANs, signal-to-interference-plus-noise ratio (SINR), and traffic priority of each sensor node in order to define the interference level. In our study, each person wears a WBAN which consists of one coordinator and multiple sensor nodes. Second, we formulate the scheduling problem as an optimization problem that maximizes the number of concurrent transmissions in multiple WBANs in each timeslot. Finally, we propose the ITLS algorithm based on the optimization problem. We also propose a condition to determine the interference level of each WBAN for each channel or timeslot. The interference level is the main factor for scheduling transmissions at each WBAN without interfering with its neighbors. As a result, the proposed ITLS algorithm can reduce inter-WBAN interference and achieve higher performance.

The rest of this paper is organized as follows: In the following section, we review the existing interference mitigation schemes for WBANs. In Section 3, the ITLS algorithm is presented and discussed. In Section 4, we analyze the proposed algorithm in terms of network throughput and spatial reuse factor. In Section 5, the performance of the proposed algorithm is evaluated via a computer simulation and compared with the conventional scheme. Finally, the paper is concluded in Section 6.

## 2. Related Works

In general, the interference problems of wireless networks have been studied and modeled as an interference graph [18]. For example, SINR at a node can be used to indicate the possibility of interference. In wireless sensor networks, interference can be mitigated by efficient scheduling as shown in the two examples of the link schedule optimization in reference [19] and TDMA scheduling algorithm in reference [20].

Inter-WBAN interference has also been studied in terms of performance degradation and some interference mitigation schemes have been developed [14]. Currently, the IEEE 802.15.6 standard provides not only the PHY and MAC specifications but also the mechanisms for coexistence and interference between WBANs [2]. In the standard, the three mechanisms of beacon shifting, channel hopping, and active superframe interleaving can be used to mitigate interference between WBANs. To further investigate WBAN performance in the context of inter-WBAN interference, some existing works have focused on analyzing the degradation of system performance, such as packet loss and delay latency [10]. Furthermore, channel scheduling at the MAC layer is exploited because it is regarded as the most effective technique for minimizing inter-WBAN interference in terms of system throughput. That is, inter-WBAN scheduling [21] focuses on WBAN traffic in coexistence scenarios; however, the dynamic environment is not considered and latency is increased. Other TDMA scheduling algorithms include dynamic resource allocation (DRA) [22] and adaptive internetwork interference mitigation (AIM) [23]. In DRA and AIM, the interfered nodes transmit signals orthogonally for interference avoidance. Another scheduling method at the MAC layer is the hybrid approach of carrier sense multiple access with collision avoidance (CSMA/CA) and TDMA for interference mitigation [24]. This method achieves high throughput, but does not guarantee quality of service (QoS). Graph coloring technique is also a solution to the scheduling problem, which has been extensively researched in wireless sensor networks for interference mitigation between sensor nodes. In reference [25], the random incomplete coloring (RIC) algorithm is applied to multiple WBANs, where each WBAN is randomly colored, thus resulting in the improvement of spatial reuse and throughput. In reference [26], the interfered WBANs are grouped as a cluster, and then a coloring technique is applied to achieve better performance. In clique-based WBAN scheduling (CBWS) [27], the clique-based graph among the interfered WBANs is introduced and the random coloring algorithm is applied for scheduling. In this scheme, the WBAN sensors are formed for several cliques, and then the sensor nodes can be allocated into timeslots by the random coloring algorithm. This scheduling algorithm does not consider whether a WBAN is severely interfered by neighboring WBANs. An interference-aware MAC [28] was also introduced to mitigate interference in healthcare monitoring. In reference [13], a QoS MAC protocol for multiple WBANs overcomes the inter-WBAN interference caused by WBAN mobility. In addition, power control schemes in WBANs are used to manage inter-WBAN interference by reducing transmission power [15,16,17]. Although some self-learning techniques can improve system throughput, the schemes do not support QoS and have a long convergence time. For WBANs capable of cognitive radio, the inter-WBAN interference problem should also be considered in the design because secondary nodes should sense the channels and switch the working channel appropriately in order to avoid interference [29]. Other existing works for inter-WBAN interference mitigation are summarized and discussed in more detail in reference [14].

It should be noted that there exist some limitations in the existing schemes. The existing link scheduling algorithms for WBANs only focus on the interference at each WBAN and on the channel status [21,24,25,27]. However, it is necessary to consider the number of interfered nodes as well as the number of interfering WBANs because the interfered nodes cause data loss at the coordinator. Some works have focused on shifting or scheduling the transmission time without taking the traffic priority into consideration [21,24,25,26,27]. In fact, the traffic priority of sensor nodes is important because different on-body sensor nodes collect different types of data. Data can be characterized in accordance with data rate, packet length, and transmission time [1]. Therefore, based on the traffic priority, the transmission links from sensor nodes to the coordinator need to be assigned to the superframe as follows: if high and low priority nodes need to transmit at the same time, the scheduling algorithm allows the high priority node to use the link to the coordinator to access the medium, whereas the low priority node has to wait.

## 3. Interference-Aware Traffic-Priority-Based Link Scheduling

In this section, we present our proposed link scheduling algorithm for a network of *n* WBANs. The network model is introduced first, and the problem of link scheduling in multiple WBANs is formulated. Then, the proposed algorithm is presented in detail with examples, and it is compared to conventional algorithms qualitatively.

### 3.1. Network Model and Interference Graph Generation

In multiple WBANs, interference can occur if more than two neighboring nodes transmit at the same timeslot and a receiver exists in the overlapped area of their transmission ranges. That is, an interfered node is the node existing within the overlapped area of the transmission ranges.

In our algorithm, the transmission of all WBANs is successfully scheduled into the superframe, where the superframe length is divided into *T* timeslots. We also assume that a WBAN is formed as a simple star topology consisting of a coordinator and a group of wireless sensor nodes, and the WBAN topology does not change during one superframe.

We consider a network with *n* WBANs labeled *B*_1_, *B*_2_, …, *B_n_* that exist within the interference range of each other. The communication coverage of each WBAN is modeled as a circle of radius *r*, where the coordinator is placed at the center and *m* sensor nodes are placed within the circle. The sensor nodes carry the vital signals of the human body, which are classified into eight different categories as per the IEEE 802.15.6 standard [2], as listed in Table 2. Each WBAN can be described by a list of sensors with traffic priority. The *j*-th sensor in *B_i_* is represented as s*_i,j_*, and the traffic priority of the *j-*th sensor in *B_i_* is denoted as *p_i,j_*, where 1 ≤ *j* ≤ *m*. Assume that for each type of traffic priority, the length of the generated packets is different; therefore, the transmission time of sensor node s*_i,j_* at priority *p_i,j_* is represented as *t_i,j_* = *u*(*p_i,j_*)/*b*(*p_i,j_*), where *u*(*p_i,j_*) is the packet length and *b*(*p_i,j_*) is the data rate of priority *p_i,j_*. For each WBAN, the coordinator schedules the transmission of the sensor nodes using the TDMA scheme. The sensor nodes receive a beacon message from the coordinator, and then transmit the vital signals following the TDMA schedule.

As given in reference [30], we assume that body-to-body links are modeled by free space path loss model without shadowing, with the path loss exponent of 2. This path loss is exponentially increased with the increased distance between WBANs. The channel gain of body-to-body links is modeled as gamma distribution as in reference [31].

At every WBAN, the coordinator discovers its neighbors using distance-based interference: if the distance between two coordinators is less than the transmission range, two WBANs interfere with each other. The list of WBANs interfering with *B_i_* is represented by *L_i_* = {*j|d_i,j_* < *r*}, where *d_i,j_* is the distance between *B_i_* and *B_j_* and *r* is the transmission range. Given *n* WBANs, the interference graph is represented by graph *G* = (*V*, *E*); *V*(*G*) is the set of WBANs, i.e., *V*(*G*) = {*B*_1_, *B*_2_, …, *B_n_*}, and *E*(*G*) is the set of interfered links between WBANs, i.e., *E*(*G*) = {(*i*, *j*)*|i*∈*L_j_*, *j*∈*L_i_*}. In case there is no interference between WBANs, *E*(*G*) = *φ*.

At WBAN *B_i_*, the coordinator creates interfered sensor group *ISG_i_* that consists of all the sensor nodes interfered by the neighbors. The coordinator always monitors the SINR of its sensor nodes, *γ_i,j_*, and compares the SINR with the threshold SINR, *γ_th_*. In the IEEE 802.15.6 standard, the transmitted power is set as −20 dBm and the sensitivity of receiver is set at −90 dBm in the noisy environment. Because we use the free space path loss model for body-to-body links, we have evaluated the SINR of an intra-WBAN communication while increasing the number of nearby WBANs from 1 to 12. As a result, the SINR value is below 0 dBm. Therefore, in this paper, we assume that the threshold SINR is 0 dBm. If SINR at a sensor is below the predetermined threshold, the sensor can be considered as an interfered sensor. SINR at sensor *s_i,j_* in *Bi*, *γ_i,j_*, can be represented as:

(1) $$\gamma_{i,j}=\frac{P_{i,j}}{\sigma^{2}+\sum_{l\neq i}P_{l,j}}$$

where *P_i,j_* is the received signal power at *s_i,j_* (originates from the coordinator of *B_i_*), *P_l,j_* is the interference power that originates from *B_l_* (from the coordinator or any sensor) and received by the sensor *s_i,j_*, and σ^2^ is the power of additive noise. If *γ_i,j_* < *γ_th_*, sensor *s_i,j_* is listed in the interfered sensor list *ISG_i_* from the coordinator. That is, *ISG_i_* = {*s_i,j_*|*γ_i,j_* < *γ_th_*}, where 1 ≤ *j* ≤ *m*. Otherwise, sensor *s_i,j_* is added to the non-interfered sensor list *NISG_i_* from the coordinator. All WBANs create the interfered sensor list and exchange it with their neighbors at every superframe.

While considering traffic priority, we calculate the weighted interference of each sensor, *w_i,__j_*, as follows:

(2) $$w_{i,j}=\frac{\gamma_{i,j}}{\gamma_{th}}\cdot p_{i,j},j\in ISG_{i}$$

The weighted interference is used not only for calculating the interference level of each coordinator but also for obtaining the contention value (which will be presented in Section 3.3) in defining the weight constraint of each WBAN at each timeslot. Each coordinator calculates its interference level by adding the weighted interference of the sensors in *ISG_i_*, as follows:

(3) $$ww_{i}=\sum_{j\in ISG_{i}}w_{i,j}$$

The parameters and notations are listed in Table 3.

### 3.2. Problem Formulation

In a network with *n* WBANs, each of which consists of *m* sensor nodes, we assume that there are *m* × *n* orthogonal transmissions in one superframe that contains *T* timeslots. The maximum number of required timeslots is *T* = *m* × *n*. The number of received packets of *s_i,j_* at the coordinator of *B_i_* is *u_i,j_*. The total network throughput is calculated as:

(4) $$BW=\frac{\sum_{i=1}^{n}\sum_{j=1}^{m}u_{i,j}}{T}$$

In order to increase the network throughput, we aim to maximize the number of nodes that can transmit at the same timeslot. This leads to a problem of maximizing the shared timeslots among WBANs. The number of transmissions in *n* WBANs is *m* × *n*, and |*T*| is the set of timeslots in one superframe. At the *k*-th timeslot of the superframe, there are up to *n* transmissions in *n* WBANs that go from the sensor nodes to their coordinators; the scheduling decision can be represented by a vector *z_k_* = [*z_i,j,k_*, *z_l,h,k_*, …, *z_m,n,k_*], where *z_i,j,k_* = 1 if the *j*-th sensor in *B_i_* is scheduled in the *k*-th timeslot; otherwise, *z_i,j,k_* = 0. Two WBANs can share the same timeslot if they do not share the same edge (i.e., the same interference link) in the interference graph. Because of the difference in traffic priority levels, the required transmission time of each priority can be different from the others. Given the traffic priority level of each sensor node, we assume that the WBAN with high priority can occupy the channel until its transmission finishes. In order to find the maximum number of WBANs that can transmit in one timeslot, we formulate the scheduling problem as an optimization problem:

(5) $$\max\sum_{i=0}^{n}\sum_{j=1}^{m}\sum_{k=1}^{T}z_{i,j,k}t_{k}$$

In this formulation, the scheduling algorithm attempts to maximize the number of sensors that can transmit in the *k*-th timeslot. However, the network throughput is measured in data packets per second, and the objective Equation (5) is rewritten as follows:

(6) $$\max\sum_{i=1}^{n}\sum_{j=1}^{m}\sum_{k=1}^{T}\frac{z_{i,j,k}u(p_{i,j})}{t_{k}}$$

subject to:

(6a) $$z_{i,j,k}+z_{l,h,k}\leq 1|j\in ISG_{i},h\in ISG_{l},l\in L_{i}$$

(6b) $$1<z_{i,j,k}+\sum_{\begin{array}{l} l\notin L_{i}\\ h\in NISG_{l} \end{array}}z_{l,h,k}\leq T$$

(6c) $$\sum_{i=1}^{n}\sum_{j=1}^{m}z_{i,j,k}\leq n$$

(6d) $$z_{i,j,k}=[0,1]$$

(6e) $$t_{k}=\max_{i\in V}t_{i,j,k}z_{i,j,k}|z_{i,j,k}=1,\;t_{i,j,k}=\frac{u(p_{i,j})}{b(p_{i,j})}z_{i,j,k}$$

We present a traffic-priority-based scheduling algorithm for Equation (6). Because the scheduling problem has a slotted structure in the time domain, multiple transmissions from multiple WBANs can be scheduled in one timeslot. There are some constraints in Equation (6) as follows: if more than two WBANs interfere with each other, only one sensor node that belongs to the *ISGs* of two neighbors can transmit at the *k*-th timeslot; this constraint is shown in Equation (6a). The non-interfered WBANs can share the same timeslot, as indicated in Equation (6b). In order to avoid intra-WBAN interference, the constraint in Equation (6c) shows that only one sensor node of a WBAN can transmit at one timeslot. The *j*-th sensor node of the *i*-th WBAN is active at the *k*-th timeslot if *z_i,j,k_* = 1; otherwise, *z_i,j,k_* = 0, as indicated in Equation (6d). In Equation (6e), the length of the *k*-th timeslot is the longest transmission time of all the sensor nodes that are active in the *k*-th timeslot.

### 3.3. ITLS Algorithm

The step-by-step operation of the proposed ITLS algorithm is given in Algorithm 1. This algorithm executes at the coordinator of each WBAN and solves the problem in Equation (6). We use a contention value (CV) to define the weight constraint of each WBAN at each timeslot, as follows:

(7) $$CV(i,k)=\sum_{j}w_{i,j}|j\in I_{i,k}$$

where *I_i,k_* is the list of interfered sensors in the *i*-th WBAN at the *k*-th timeslot. The contention value of *B_i_* is denoted as *CV*(*i*, *k*) which is used to content the timeslot indexed by *k* in the superframe. The coordinator of *B_i_* calculates its contention value which is defined by the summation of the weighted interference values of the sensors in the *ISG_i_* as shown in Equation (7).

**Figure 2:**
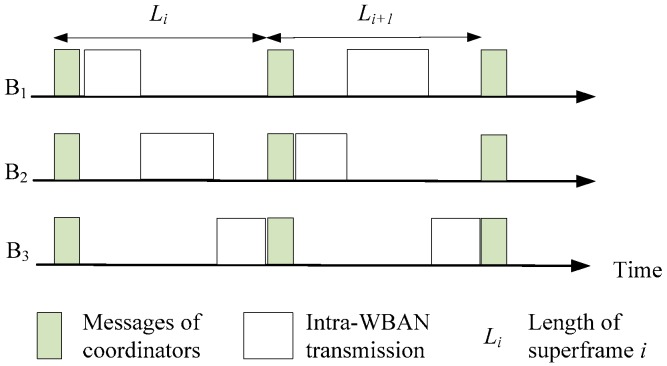
Superframe structure for WBANs showing messages of coordinators and intra-WBAN transmissions.

Each WBAN is assigned to the scheduling vector of each superframe; the scheduling vector consists of the timeslot index for each sensor node in the superframe. In Algorithm 1, at each timeslot, all WBANs calculate their contention value, as shown in line 3. If other WBANs interfere with a given WBAN, the latter exchanges its scheduling message with its neighbors before starting transmission. The superframe structure is shown in Figure 2, where the coordinators of WBANs will exchange the message with their neighbors before starting intra-WBAN communication. The interfered WBAN compares its contention value with that of the interfering WBANs (line 4). The winner in this step is the WBAN with the highest CV. The active sensor with the highest priority is chosen for scheduling (lines 5 to 7). In lines 8 to 11, the winner’s neighbors choose the sensor node to transmit at the *k*-th timeslot. In lines 12 to 15, the non-neighbors of the winner choose the sensor node to transmit at the *k*-th timeslot.

**Algorithm 1:** ITLS algorithm**Input**: *G* = (*V*, *E*), *T***Output**: *z_i,j,k_*, *t_k_*, and *K*Each WBAN creates its *L_i_*, *ISG_i_*, and *NISG_i_*;Initialize a temporal vector for *B_i_*: a temporal scheduling value for each *j*-th sensor in each *B_i_*: z*_i,j,1_* = 0; list of interfered sensor at first slot: *I_i_*_,1_ = *ISG_i_*, list of non-interfered sensor at first slot: *NI_i_*_,1_ = *NISG_i_***while** all WBANs are not scheduled  **if** *k* < *T*    calculate contention value *CV*(*i*,*k*)|∀*i*∈V as in (7)    find *CV_max_* = max(*CV*(*i*,*k*); return *j*-th sensor of *i*-th WBAN (*j*∈*I_i,k_*)    find required transmission time for *s_i,j_*, which is *t_i,j,k_*    set *z_i,j,k_* = 1; remove *j* of *I_i,k_*    update *t_k_* = *t_i,j,k_* to network    **for** each *B_l_*∈*L_i_*      return *h*-th sensor of *l*-th WBAN (*h*∈*NI_l,k_*) such that *h*-the sensor has the highest priority      set *z_l,h,k_*= 1; remove *h* of *I_l,k_*    **end for**    **for** each *B_x_*∉*L_i_*      return *y*-th sensor of *x*-th WBAN (*y*∈*I_x,k_*) such that *y*-th sensor has the highest priority      set *z_x,y,k_* = 1; remove *y* of *I_x,k_*    **end for**    update *k* = *k* + 1  **end if****end while**update *K* = *k*

In one timeslot, the high priority sensor node can occupy the channel, and more than one WBAN can transmit via the channel with low interference, thus ensuring the continuity of transmission. When the coordinator records any changes to the SINR of its sensor node, the coordinator updates scheduling for the new transmission at the end of the existing superframe. Otherwise, the scheduling is kept the same.

### 3.4. ITLS Example

**Figure 3:**
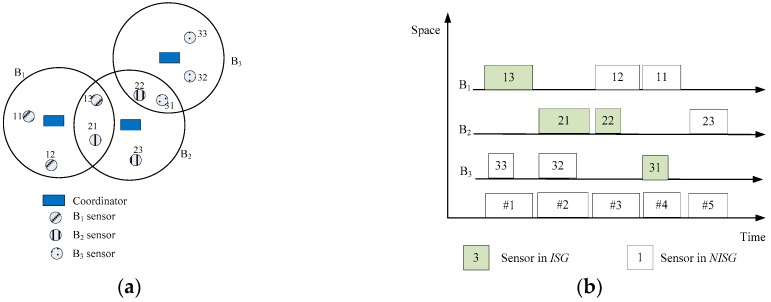
Interference-aware traffic-priority-based link scheduling (ITLS) example: (**a**) Three WBANs and their interference areas; (**b**) Timeslot assignment for network.

An ITLS example is shown in Figure 3. Assume that there are three WBANs, B_1_, B_2_, and B_3_ that work at the same time and at the same place. Three WBANs and their interference areas are shown in Figure 3a. The sensor list of each WBAN with traffic priority written in parenthesis is as follows: B_1_ = {11(1), 12(4), 13(5)}, B_2_ = {21(2), 22(1), 23(2)}, and B_3_ = {31(1), 32(2), 33(3)}. The interference areas of the WBANs consist of the sensor nodes {13, 21, 22, 31} as shown in Figure 3a. Each WBAN creates its *ISG* and *NISG* as listed in Table 4.

At the first timeslot, *B*_1_ has the highest CV (because of the priority of the interfered sensors) and *ISG*(1) = {13}; therefore, *B*_1_ occupies the channel and sensor “13” is the highest priority sensor node in *ISG*(1) that transmits data; while *NISG*(3) is {32, 33}, *B*_3_ can choose one of the sensors in its *NISG* to transmit. The scheduling decision at the first timeslot is *B*_1_(13) and *B*_3_(33). At the second timeslot, *B*_2_ has the highest CV; therefore, one of the sensor nodes in *ISG*(2) can transmit data; because *B*_3_ does not share the same edge, *B*_3_ can transmit. The scheduling decision at the second timeslot is *B*_2_(21) and *B*_3_(32). The final scheduling among WBANs is shown in Figure 3b. The total required timeslot for three WBANs is nine orthogonal transmissions, but the proposed algorithm requires five timeslots. Therefore, the spatial reuse is 9/5, and up to two sensor nodes can share the same timeslot.

### 3.5. Qualitative Comparison of Link Scheduling Algorithms

In this subsection, a qualitative comparison of link scheduling algorithms for multiple WBANs is summarized and discussed. The comparison results are summarized in Table 5.

The interference model of multiple WBANs is based on SINR or distance. A WBAN can be interfered if the transmission ranges of WBANs are overlapped and the SINR of sensor nodes is below the threshold. For example, the coordinator can detect the interfered sensor node based on SINR in DRA and AIM. Moreover, if the distance between two coordinators is less than the mutual interference range between WBANs, those WBANs are interfered as in the RIC and CBWS algorithms. In our proposed algorithm, the coordinator detects the interfered sensor nodes using the SINR model. The scheduling decision is made at the coordinator that can consider the entire WBAN or individual sensor nodes. Among the existing link scheduling algorithms, RIC schedules WBANs, whereas DRA, AIM, and CBWS schedule the sensor nodes. Scheduling all the sensor nodes in multiple WBANs is advantageous in comparison to the two-step hierarchical approach of scheduling among WBANs first and scheduling among sensor nodes within a WBAN. As a consequence, nodes can avoid collision with the transmission of other WBANs. Our proposed ITLS schedules the sensor nodes with regard to interference level and traffic priority. Among the existing link scheduling algorithms, only AIM schedules the sensor nodes based on traffic priority, whereas the others do not consider traffic priority. The existing algorithms are based on TDMA, where each sensor node is assigned to the fixed timeslot. In our proposed algorithm, the set of transmission links from the sensor nodes to the coordinator is mapped into one timeslot.

## 4. Analysis of Proposed Algorithm

In this section, we calculate the average system throughput and spatial reuse factor of the network under study. The system throughput is defined as the effective transmission per slot that counts the data transmission of all sensor nodes actually received by all the coordinators in the network. The spatial reuse factor is defined as the average number of sensor nodes that share the same timeslot.

Given a network with *n* WBANs, *V*(*G*) = {*B*_1_, *B*_2_, …, B_n_} is the set of WBANs. The total number of sensor nodes in the network is *m* × *n*. Let *BW* denote the system throughput in the network, which is the sum of data rates received by all the coordinators. Throughput is calculated as follows:

(8) $$BW=\frac{\sum_{i=1}^{n}\sum_{j=1}^{m}u_{i,j}}{T}$$

where *u_i,j_* is the number of received packets of *s_i,j_* in *B_i_*.

The required transmission time for the network is calculated as follows:

(9) $$T=\sum_{i=1}^{n}T_{i}$$

where *T_i_* is the required transmission time of *B_i_*.

The probability that *k* WBANs are interfering is denoted by:

(10) $$P_{k}=\left(\begin{array}{l} n\\ k \end{array}\right)p^{k}{\left(1-p\right)}^{n-k}$$

where *p* is the probability that other WBANs interfere with *k* WBANs.

In a group of *k* WBANs, each WBAN creates its *ISG* by considering the SINR of the sensor nodes. The set of interfered sensors in *k* interfering WBANs is:

(11) $$SI_{k}=ISG_{i}\cup ISG_{l}|i\in L_{l},l\in L_{i},1\leq i,l\leq k$$

Therefore, the required transmission time of *SI_k_* is given by:

(12) $$TS_{k}=\sum_{i\in SI_{k}}\sum_{j\in ISG_{i}}t_{i,j}$$

Assume that the maximum degree of the network is *k*, and the required transmission time for *k* interfered WBANs is *T_k_*. Because the non-interfered nodes and two-hop neighbors can share the same timeslot, the required transmission time for the non-interfered nodes is *T_nk_*.

There exists the *i*-th WBAN with the maximum degree of the network. The required transmission time for this WBAN can be calculated by:

(13) $$T_{i}=\sum_{x\in SI_{k}}\sum_{j\in ISG_{x}}t_{x,j}+\sum_{j\in NISG_{i}}t_{i,j}$$

The transmission time for *k* interfered WBANs is:

(14) $$T_{k}=T_{i}$$

The total transmission time of the network is calculated by:

(15a) $$T=\max\{T_{k},T_{nk}\}$$

and

(15b) $$T=T_{k},\;\mathrm{if}\;k>\frac{n}{2}$$

The waiting time of the *i*-th WBAN is calculated as follows:

(16) $$WT_{i}=T-T_{i}$$

For each timeslot, the length of a timeslot is the length of the highest traffic data to finish its transmission. Therefore, it can be calculated as in Equation (6e):

(17) $$t=\max_{i\in V}t_{i,j}z_{i,j,a}|z_{i,j,a}=1,t_{i,j}=\frac{u(p_{i,j})}{b(p_{i,j})}$$

The spatial reuse factor is defined as the average number of sensor nodes that share the same timeslot, which is calculated as follows:

(18) $$\sigma =\frac{m\times n\times t_{s}}{T}=\frac{m\times n}{T_{k}}$$

The average network throughput is defined as the effective transmission per slot that counts the data transmission of all sensor nodes actually received by all the network coordinators. Therefore, it can be calculated as follows:

(19) $$BW=\frac{\sum_{i=1}^{n}\sum_{j=1}^{m}u_{i,j}}{T_{k}}$$

## 5. Performance Evaluation

In this section, the performance of the proposed ITLS is evaluated via MATLAB simulation and compared with the conventional algorithm AIM [23]. Note here that AIM is selected for comparison because AIM considers traffic priority while allocating sensor nodes for each interfered WBAN. Interested readers can refer to reference [23] for more details.

### 5.1. Simulation Environment

A typical example of realistic scenarios with practical WBAN applications is health monitoring within a hospital where there are many patients wearing a WBAN [8,9]. In such a scenario, the inter-WBAN interference may occur because data can be transmitted and received by multiple WBANs at the same time. In order to reflect the realistic scenario with the practical WBAN applications for healthcare monitoring, we consider a combined network consisting of many WBANs as in references [8,9] where each WBAN has some biomedical sensor nodes. Furthermore, it should be noted that WBANs are assumed to be mobile in our simulation study.

We consider a simulation area of 10 m × 10 m while varying the number of coexisting WBANs as in reference [28]. Initially, all WBANs are uniformly deployed. The typical star topology is used for each WBAN, in which six biomedical sensor nodes are wirelessly connected to one coordinator as in reference [3]. In our simulation, the coordinator is deployed at the center of a WBAN and the sensor nodes are randomly deployed within the transmission range of 2 m [3]. The transmit power and receiver sensitivity are set as −20 dBm and −90 dBm, respectively, as specified in the IEEE 802.15.6 standard. We use the free space path loss model with a path loss exponent of 2 for intra-WBAN communication. In medical applications, the WBAN traffic generated at sensor nodes is categorized into different levels of priority [7]. More specifically, we set the traffic priority at each sensor according to the IEEE 802.15.6 standard as in Table 2. For satisfying the requirement of different kinds of practical WBAN applications, the packet size can be varied according to the traffic priority shown in Table 2. In our simulation study, it is assumed that the packet size is linearly increased with the increased priority level from 50 bytes (at priority level 1) to 350 bytes (at priority level 7). In order to control the traffic in our simulation, the packet outgoing rate of each node is varied, which is 1, 2, 3, 4, 8, and 16 packets per second. The transmission rate is defined as 240 kbps according to IEEE 802.15.6. For modeling the mobility of WBANs, the typical random waypoint model is used as in reference [28], in which the node speed is less than 2 m/s and the pause time is 30 s. As a result, the inter-WBAN connectivity is dynamically changed during the simulation time. In our simulation, two factors are considered: the number of WBANs and packet outgoing rate at each sensor node. We obtained the average results of the simulation after 20 iterations. The detailed settings of simulation parameters are listed in Table 6.

### 5.2. Simulation Results and Discussion

#### 5.2.1. Packet Delivery Ratio

**Figure 4:**
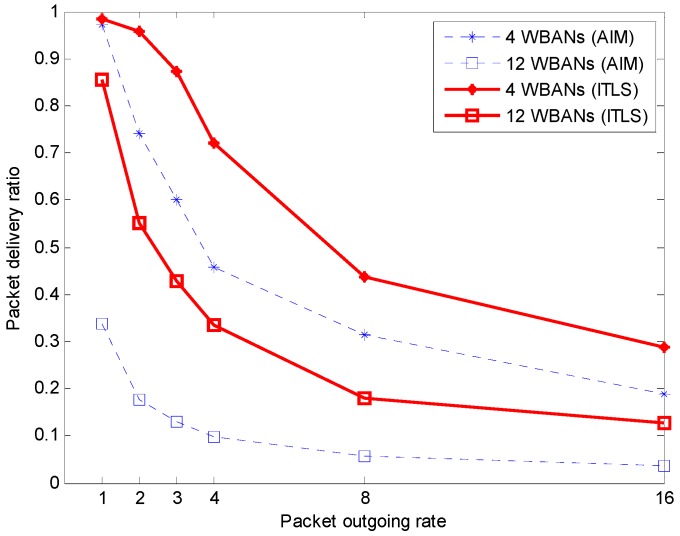
Packet delivery ratio.

At each WBAN, the packet delivery ratio (PDR) is the ratio of successfully received packets at the coordinator to the total number of generated packets at the sensors of the *i*-th WBAN. Figure 4 shows the PDR of the proposed algorithm and compares it with that of the conventional AIM. The PDR decreases when the number of WBANs increases. If the packet outgoing rate of the sensor nodes increases, the PDR decreases. Also, the PDR decreases with increased number of WBANs. As shown in Figure 4, the proposed ITLS always achieves higher PDR than AIM. This is mainly because the schedule of a superframe is shared among WBANs in our algorithm. That is, the first available timeslot is assigned to only the sensor node with the highest priority or the longest packet size. Some packets generated by sensor nodes may be dropped inevitably because of long waiting time. In addition, the two-hop neighbors can reuse the same timeslot, resulting in increased network throughput.

#### 5.2.2. Spatial Reuse Factor

**Figure 5:**
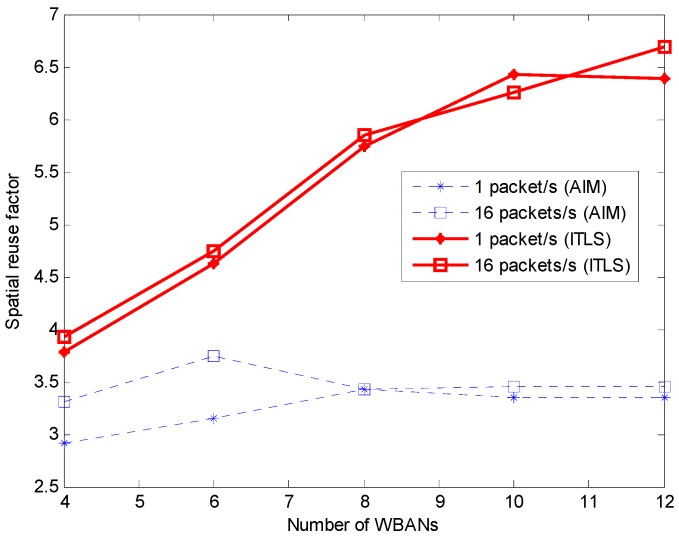
Spatial reuse factor.

In our study, the spatial reuse factor is defined as the average number of sensor nodes that share the same timeslot. Figure 5 shows that the proposed ITLS achieves higher spatial reuse than AIM. More sensor nodes can transmit to their coordinators in the same timeslot without interfering with their neighbors. As shown in the figure, the spatial reuse factor depends on the number of WBANs for both algorithms. Our proposed ITLS has a higher spatial reuse factor than AIM because the nodes in *NISG* can be transmitted at the same timeslot of *ISG* if WBANs are not interfered. Notice again that the schedule of a superframe is shared among WBANs in our algorithm. Therefore, the two-hop neighboring WBANs can reuse the timeslot, thus increasing the spatial reuse factor.

#### 5.2.3. System Throughput

**Figure 6:**
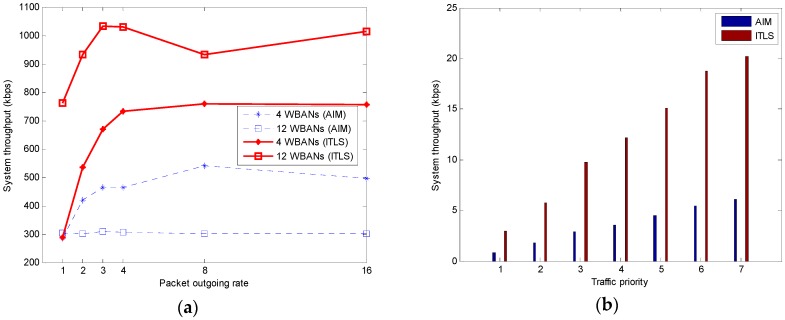
(**a**) System throughput; (**b**) System throughput with regards to traffic priority.

System throughput is defined as the effective transmission per slot that counts the data transmission of all sensor nodes actually received by all network coordinators. This metric is measured in bps (bit per second). The system throughput is shown in Figure 6. As Figure 6a shows, the proposed ITLS achieves higher system throughput than AIM because of high spatial reuse. It should be noted that the first available timeslot is assigned to only the sensor node with the highest priority or the longest packet size in the schedule of a superframe shared among WBANs. Furthermore, the two-hop neighbors can reuse the timeslot. This results in increased network throughput.

We also consider the throughput of each type of traffic priority in the case of 12 WBANs and 16 packets per second. The results shown in Figure 6b indicate that the traffic priority of our proposed ITLS depends on traffic priority. The nodes with the highest traffic priority can access the channel, and the throughput is higher than that of the lower traffic priority. Due to the assumption of longer packet size for higher traffic priority, it is clearly shown in Figure 6b that, in the same interference scenario, the high priority nodes have higher system throughput than the low priority nodes.

#### 5.2.4. Average Packet Delay

**Figure 7:**
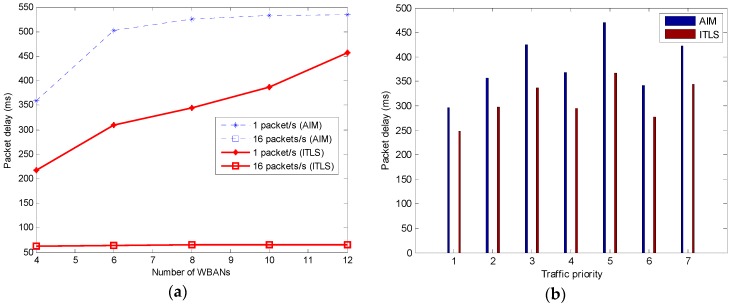
(**a**) Average packet delay; (**b**) Average packet delay in terms of traffic priority.

Average packet delay is the time between the generation of a packet at a sensor node and the reception of the packet at the coordinator. In Figure 7, it is observed that network traffic and node density affect to the average packet delay. Compared with AIM, the proposed ITLS has lower delay when the packet outgoing rate is low. Note that the low packet outgoing rate means low network traffic. However, with high traffic, the results of both algorithms become similar, as shown in Figure 7a. Figure 7b shows that our algorithm has lower delay then AIM for every type of traffic priority. This is mainly due to the fact that, in the proposed ITLS, the schedule of a superframe is shared among WBANs and the first available timeslot is assigned to only the sensor with the highest priority or the longest packet size. Moreover, the two-hop neighbors can reuse the timeslot in our algorithm. As a result, the packet delay is decreased.

## 6. Conclusions

In this paper, a novel link scheduling algorithm for multiple WBANs has been proposed not only to mitigate inter-WBAN interference but also to increase the spatial reuse of channels. By taking traffic priority, packet length, and the number of interfered sensor nodes into consideration, the proposed ITLS achieves high spatial reuse and high throughput. In ITLS, the schedule of a superframe is shared among WBANs and the first available timeslot is assigned to only the sensor with the highest priority or the longest packet size. Furthermore, both of the two-hop neighbors can transmit at the same timeslot. ITLS also ensures that high traffic priority has more opportunities to access the channel. Our extensive performance study shows that the proposed ITLS significantly increases spatial reuse and network throughput with lower delay by mitigating inter-WBAN interference. For our future work, we will consider transmission power control and human mobility in the scenarios of densely deployed WBANs, such as environments for healthcare applications.

## Figures and Tables

**Table 1:** Parameters of wireless body area networks (WBAN) applications [11].

Application Type	Data Rate	Latency
ECG ^1^ (12 leads)	288 kbps	250 ms ^4^
ECG (6 leads)	71 kbps	250 ms
EMG ^2^	320 kbps	250 ms
EEG ^3^ (12 leads)	432 kbps	250 ms
Hearing aid	200 kbps	250 ms
Video/med imaging	10 Mbps	100 ms
Voice	50–100 kbps	100 ms
Audio	1 Mbps	100 ms

^1^ Electrocardiograms; ^2^ Electromyography; ^3^ Electroencephalogram; ^4^ millisecond.

**Table 2:** Traffic priority in WBANs [2].

Traffic Priority (*TP*)	Traffic Designation
0 (Lowest)	Background (BK)
1	Best effort (BE)
2	Excellent effort (EE)
3	Video (VI)
4	Voice (VO)
5	Medical data or network control
6	High-priority medical data or network control
7 (Highest)	Emergency or medical implant event report

**Table 3:** Parameters and notations.

Notation	Description
*B_i_*	*i*-th WBAN
*s_i,j_*	*j*-th sensor in *B_i_* (*s_i,_*_0_ is the coordinator of *B_i_*)
*r*	Transmission range
*n*	Number of WBANs in a network
*m*	Number of sensors in a WBAN
*p_i,__j_*	Traffic priority of *s_i,j_*
*t_i,j_*	Transmission time of *j*-th sensor in *B_i_*
*u(p_i,j_)*	Packet length of *s_i,j_*
*b(p_i,j_)*	Data rate of *s_i__,j_*
*γ_th_*	Threshold level of received power
*γ_i,__j_*	SINR at *s_i,j_*
*L_i_*	List of WBANs interfering with *B_i_*
*I_i,k_*	List of interfered sensors in *B_i_* at *k*-th timeslot
*t_i,j__,k_*	Transmission time of *j*-th sensor in *B_i_* at *k*-th timeslot
*ISG_i_*	Interfered sensors group in *B_i_*
*NISG_i_*	Non-interfered sensors group in *B_i_*
*w_i,__j_*	Weighted interference of *s_i,j_*, *j*∈*ISG_i_*
*ww_i_*	Weighted interference of *B_i_*

**Table 4:** Example list of ISG and NISG.

WBAN	ISG	NISG
**B_1_**	{13}	{11, 12}
**B_2_**	{21, 22}	{23}
**B_3_**	{31}	{32, 33}

**Table 5:** Qualitative comparison of link scheduling algorithms.

Algorithm	Metric for Interference Graph	Scheduling Decision	Traffic Priority	Scheduling Policy
DRA ^1^ [22]	SINR at sensor nodes	Sensor node	No	Orthogonally scheduling
AIM ^2^ [23]	SINR at sensor nodes	Sensor node	Yes	TDMA ^5^
RIC ^3^ [25]	Distance	WBAN	No	TDMA
CBWS ^4^ [27]	Distance	Group of sensors	No	TDMA
Proposed ILTS	SINR at sensor nodes and distance	Sensor node	Yes	STDMA ^6^

^1^ Dynamic Resource Allocation; ^2^ Adaptive Internetwork Interference Mitigation; ^3^ Random Incomplete Coloring; ^4^ Clique-Based WBAN Scheduling; ^5^ Time Division Multiple Access; ^6^ Space-Time Division Multiple Access.

**Table 6:** Simulation parameters.

Parameter	Value
Simulation area	10 m × 10 m
WBAN topology	Star topology consisting of one coordinator and six sensor nodes
Number of WBANs	4, 6, 8, 10, 12
Transmission range	2 m
Priority level	1 to 7
Transmission rate	240 kbps
Receiver sensitivity	−90 dBm
Transmit power	−20 dBm
Mobility model	Random waypoint model (speed 0~2 m/s, pause time 30 s)
Packet size	50 bytes (at priority level 1)
	350 bytes (at priority level 7)
Channel model	Free space path loss model with path loss component of 2
Simulation time	600 s
Number of iterations	20
Frequency	2.4 GHz

## References

[1] Otto C., Milenkovic A., Sanders C., Jovanov E. (2006). System Architecture of a Wireless Body Area Sensor Network for Ubiquitous Health Monitoring. J. Mob. Multimed..

[2] Astrin A. (2012). IEEE Standard for Local and Metropolitan Area Networks Part 15.6: Wireless Body Area Networks.

[3] Movassaghi S., Abolhasan M., Lipman J., Smith D., Jamalipour A. (2014). Wireless Body Area Networks: A Survey. IEEE Commun. Surv. Tutor..

[4] (2006). IEEE Standard for Information Technology—Local and Metropolitan Area Networks—Specific Requirements—Part 15.4: Wireless Medium Access Control (MAC) and Physical Layer (PHY) Specifications for Low Rate Wireless Personal Area Networks (WPANs).

[5] Tobón D.P., Falk T.H., Maier M. (2013). Context Awareness in WBANs: A Survey on Medical and Non-Medical Applications. IEEE Wirel. Commun..

[6] Khan J.Y., Yuce M.R., Bulger G., Harding B. (2012). Wireless Body Area Network (WBAN) Design Techniques and Performance Evaluation. J. Med. Syst..

[7] Ullah S., Kjan P., Ullah N., Saleem S., Higgins H., Kwak K.S. (2009). A Review of Wireless Body Area Networks for Medical Applications. Int. J. Commun. Netw. Syst. Sci..

[8] Yuce M.R. (2010). Implementation of Wireless Body Area Networks for Healthcare Sys December 2016tems. Sens. Actuators A Phys..

[9] Misra S., Mahapatro J., Mahadevappa M., Islam N. (2014). Random Room Mobility Model and Extra-wireless Body Area Network Communication in Hospital Buildings. IET Netw..

[10] De Silva B., Natarajan A., Motani M. Inter-User Interference in Body Sensor Networks: Preliminary Investigation and an Infrastructure-based Solution. Proceedings of the Sixth International Workshop on Wearable and Implantable Body Sensor Networks.

[11] Patel M., Wang J. (2010). Applications, Challenges, and Prospective in Emerging Body Area Networking Technologies. IEEE Wirel. Commun. Mag..

[12] Anjum I., Alam N., Razzaque M.A., Hassan M.M., Alamri A. (2013). Traffic Priority and Load Adaptive MAC Protocol for QoS Provisioning in Body Sensor Networks. Int. J. Distrib. Sens. Netw..

[13] Cheng S., Huang C., Tu C. (2011). RACOON: A Multiuser QoS Design for Mobile Wireless Body Area Networks. J. Med. Syst..

[14] Le T.T., Moh S. (2015). Interference Mitigation Schemes for Wireless Body Area Sensor Networks: A Comparative Survey. Sensors.

[15] Kazemi R., Vesilo R., Dutkiewicz E., Gengfa F. Inter-Network Interference Mitigation in Wireless Body Area Networks using Power Control Games. Proceedings of the International Symposium on Communications and Information Technologies (ISCIT).

[16] Kazemi R., Vesilo R., Dutkiewicz E., Liu R.P. Reinforcement Learning in Power Control Games for Internetwork Interference Mitigation in Wireless Body Area Networks. Proceedings of the 2012 International Symposium on Communications and Information Technologies (ISCIT).

[17] Kazemi R., Vesilo R., Dutkiewicz E. A Novel Genetic-Fuzzy Power Controller with Feedback for Interference Mitigation in Wireless Body Area Networks. Proceedings of the 2011 IEEE Conference on Vehicular Technology.

[18] Cardieri P. (2010). Modeling Interference in Wireless Ad hoc Networks. IEEE Commun. Surv. Tutor..

[19] Madan R., Cui S., Lall S., Goldsmith A.J. (2007). Modeling and Optimization of Transmission Schemes in Energy-constrained Wireless Sensor Networks. IEEE/ACM Trans. Netw..

[20] Ergen S.C., Varaiya P. (2010). TDMA Scheduling Algorithms for Wireless Sensor Networks. Wirel. Netw..

[21] Jamthe A., Mishra A., Agrawal D.P. Scheduling Schemes for Interference Suppression in Healthcare Sensor Networks. Proceedings of the IEEE International Conference on Communications (ICC).

[22] Movassaghi S., Abolhasan M., Smith D. Smart Spectrum Allocation for Interference Mitigation in Wireless Body Area Networks. Proceedings of the IEEE International Conference on Communications (ICC).

[23] Movassaghi S., Abolhasan M., Smith D., Jamalipour A. AIM: Adaptive Internetwork Interference Mitigation Amongst Co-existing Wireless Body Area Networks. Proceedings of IEEE Global Communications Conference (GLOBECOM).

[24] Kim E.-J., Youm S., Shon T., Kang C.-H. (2013). Asynchronous Inter-network Interference Avoidance for Wireless Body Area Networks. J. Supercomput..

[25] Cheng S.H., Huang C.Y. (2013). Coloring-Based Inter-WBAN Scheduling for Mobile Wireless Body Area Networks. IEEE Trans. Parallel Distrib. Syst..

[26] Movassaghi S., Abolhasan M., Smith D. Cooperative Scheduling with Graph Coloring for Interference Mitigation in Wireless Body Area Networks. Proceedings of the IEEE Wireless Communications and Networking Conference.

[27] Xie Z., Huang G., He J., Zhang Y. (2014). A Clique-Based WBAN Scheduling for Mobile Wireless Body Area Networks. Procedia Comput. Sci..

[28] Mahapatro J., Misra S., Mahadevappa M., Islam N. (2015). Interference-aware MAC Scheduling and Admission Control for Multiple Mobile WBANs used in Healthcare Monitoring. Int. J. Commun. Syst..

[29] Bhandari S., Moh S. (2015). A Survey of Mac Protocols for Cognitive Radio Body Area Networks. Sensors.

[30] Cotton S.L., D’Errico R., Oestges C. (2014). A Review of Radio Channel Models for Body Centric Communications. Radio Sci..

[31] Hu Z.H., Nechayev Y., Hall P. Measurements and Statistical Analysis of the Transmission Channel between Two Wireless Body Area Networks at 2.45 GHz and 5.8 GHz. Proceedings of the ICECom.

